# Photochemical Synthesis of Nanosheet Tin Di/Sulfide with Sunlight Response on Water Pollutant Degradation

**DOI:** 10.3390/nano9020264

**Published:** 2019-02-14

**Authors:** Juan Matmin, Mohamad Azani Jalani, Hazwanee Osman, Qistina Omar, NorulNazilah Ab’lah, Kelimah Elong, Muhd Firdaus Kasim

**Affiliations:** 1 Centre of Foundation Studies UiTM, Universiti Teknologi MARA (UiTM), Cawangan Selangor, Kampus Dengkil, Dengkil 43800, Selangor, Malaysia; hazwanee@salam.uitm.edu.my (H.O.); qistina71@salam.uitm.edu.my (Q.O.); nazlila24@salam.uitm.edu.my (N.A.); 2 Kolej PERMATA Insan, Universiti Sains Islam Malaysia (USIM), Kompleks PERMATA Insan, Bandar, Baru Nilai, Nilai 71800, Negeri Sembilan, Malaysia; mazani@usim.edu.my; 3 Centre for Nanomaterials Research, Institute of Science, Universiti Teknologi MARA (UiTM), Level 3, Block C, Shah Alam 40450, Selangor, Malaysia; kelimah0907@salam.uitm.edu.my (K.E.); muhdfir@salam.uitm.edu.my (M.F.K.)

**Keywords:** photochemical synthesis, two-dimensional materials, tin di/sulfide, light-assisted synthesis, sunlight response, photo-degradation, nanosheets morphology, methylene blue, water pollutant

## Abstract

The photochemical synthesis of two-dimensional (2D) nanostructured from semiconductor materials is unique and challenging. We report, for the first time, the photochemical synthesis of 2D tin di/sulfide (PS-SnS_2-x_, x = 0 or 1) from thioacetamide (TAA) and tin (IV) chloride in an aqueous system. The synthesized PS-SnS_2-x_ were characterized by X-ray diffraction (XRD), energy dispersive X-ray spectroscopy (EDX), field emission scanning electron microscopy (FESEM), transmission electron microscopy (TEM), a particle size distribution analyzer, X-ray photoelectron spectroscopy (XPS), Fourier-transform infrared spectroscopy (FTIR), thermal analysis, UV–Vis diffuse reflectance spectroscopy (DR UV–Vis), and photoluminescence (PL) spectroscopy. In this study, the PS-SnS_2-x_ showed hexagonally closed-packed crystals having nanosheets morphology with the average size of 870 nm. Furthermore, the nanosheets PS-SnS_2-x_ demonstrated reusable photo-degradation of methylene blue (MB) dye as a water pollutant, owing to the stable electronic conducting properties with estimated bandgap (E_g_) at ~2.5 eV. Importantly, the study provides a green protocol by using photochemical synthesis to produce 2D nanosheets of semiconductor materials showing photo-degradation activity under sunlight response.

## 1. Introduction

Over the last decades, the concerns for global warming and climate change have paved the way to the establishment of green synthesis with a highlight on environmentally friendly preparation of advanced nanomaterials [1,2,3]. Among the available methods, photochemical synthesis is pivotal, since its makes use of light as a clean, safe, cost-efficient and traceless reagent, as well as a promising renewable energy resource [4]. In comparison to traditional thermo-derived methods, photochemical synthesis is comparatively workable in mild conditions and demonstrates controllable kinetics features [5]. Significantly, the photochemical synthesis is progressively introduced in preparing nanomaterials from noble metals (Ag, Pd, Au, Pt) nanoparticles [5,6,7] or one-dimensional (1D) architectures, such as ZnS-carbon nanotubes [8], Au nanorods [9], and precious metals (Au and Ag) nanowires [10]. Despite these advantages, there are only a few reports on two-dimensional (2D) structures prepared using photochemical approaches.

In recent years, preparation of 2D nanomaterials by photochemical synthesis has been in high demand due to their impressive electronic properties and unique surface chemistry as well as beneficial quantum-size effect [11]. In this technique, light irradiation is used to good advantage to reduce metal ion precursors and promote the formation of 2D nanostructures. For example, Huang et al. synthesized 2D Au@TiO_2_ nanocomposites showing platelet-like morphology with the crucial help of the semiconductor colloidal TiO_2_ to initiate and stabilize the reaction [12]. In the more recent example, Bharat and his co-workers demonstrated the synthesis of 2D Au nanodots on α-Fe_2_O_3_@reduced graphene oxide (RGO) for hetero-nanostructured composites [13]. Briefly, they utilize chlorophyll under sunlight irradiation to stimulate a rapid reduction of Au^3+^ ions to Au° metallic nanodots on α-Fe_2_O_3_@RGO surface within 30 min [13]. In another way, photochemical synthesis had provided greener approaches to develop nanohybrid semiconductor materials such as Au@graphene and Au@metal oxide (TiO_2_, ZnO) [5,14]. Typically, the conventional methods compromise from the vital association of light-absorbing additives such as precious metals (Au and Ag), organic dyes (chlorophyll), or co-precipitation of semiconductor metal oxides (TiO_2_) which face the drawbacks of high cost and non-reusability problems [12,13,14,15]. Unfortunately, these hybrids and composites designed of semiconductor nanomaterials give insufficient sunlight response for photocatalytic activity [16,17]. Therefore, the photochemical synthesis of materials from non-hybrid/composites displaying sunlight response as a photocatalyst is urgently required. 

Today, the most promising 2D nanomaterials with sunlight response are from semiconductor materials having nanosheet structures, giving excellent electronic properties, sizable bandgaps and stable charge transfer applications [18]. However, up to now, photochemical synthesis of 2D semiconductor nanomaterials from non-hybrid/composites systems without the use of light-absorbing additives has been rare and poorly reported [19,20,21]. To the best of our knowledge, there are still no reports on the photochemical synthesis of 2D semiconducting nanomaterials from tin di/sulfide (SnS_2-x_, x = 0 or 1). Herein, we successfully prepared SnS_2-x_ as a semiconductor material having nanosheet morphology from photochemical synthesis, without the use of light-absorbing additives. Furthermore, the prepared SnS_2-x_ were used in photo-degradation of methylene blue (MB) dye with good reusability under sunlight response free from harsh oxidizing or reducing reagents. Alternatively, this strategy had presented cost-efficient usage of light in a water medium, as well as the simplicity of the process which makes the whole process a truly green synthesis.

## 2. Materials and Methods 

### 2.1. Photochemical Synthesis of Tin Di/Sulfide (PS-SnS_2-x_)

All of the starting materials are purchased from analytical grade reagents. Deionized water was used as the medium throughout the preparatory works especially during the dilution of the solution. Conventional chemicals of tin (IV) chloride pentahydrate (SnCl_4_.5H_2_O, 98%, Sigma Aldrich, St. Louis, M.O., USA), thioacetamide (TAA, 99%, Acros, N.J., USA), isopropyl alcohol (Tedia, Fairfield, C.T., USA), ethanol (99%, Fisher Chemicals, N.H., USA), and hydrochloric acid (HCl, 36.5–38.0%, J.T. Baker, A.C.S. Reagen, P.A., USA) were purchased from local suppliers. In a typical photochemical synthesis, 1.28 mmoL of SnCl_4_.5H_2_O and 4.39 mmoL of TAA were dissolved in 30 mL isopropyl alcohol in a perfectly screw-capped test tube. After 30 min of vigorous stirring, the solution was transferred to another clean test tube, before the collected precipitates were centrifuged and rinsed with large amounts of deionized water in ethanol to give a golden colored precipitate. Subsequently, the precipitate was thoroughly washed with 0.5 ml of HCl (37% *w*/*w*) and left for overnight. The solid samples were later transferred in an isolated closed-system under continuous halogen-lamp irradiation (90 W) with constant stirring for 8 h. Finally, the sample was dried under vacuum for overnight to give a pale gold solid. For the convenience of description, the collected solid was referred to as “PS-SnS_2-x_” with x is either 0 or 1. 

### 2.2. Characterization 

The crystalline state of PS-SnS_2-x_ was evaluated using X-ray diffractometer (XRD, Ultima IV, Rigaku Corporation, Japan) with Cu Kα radiation (λ = 1.54 Å) at 30 kV voltage and 15 mA current. The scanning speed was set at 3°/min and in the range between 3° and 80° (2θ). The morphology of the sample was observed using field emission scanning electron microscopy (FESEM, JSM-6700F, JEOL, Tokyo, Japan), attached with energy dispersive X-ray spectroscopy (FESEM-EDX), operated at 3.0 kV to determine the elemental composition of the samples. Prior FESEM analysis, the sample was pre-coated with aluminum (Al) at 10^−1^ mbar using a Bio-Rad system. Transmission electron microscopy (TEM) micrographs of the samples were recorded by a JEM-2100F Electron Microscope (JEOL, Tokyo, Japan) at 160 kV accelerated voltage. The samples were ultrasonically dispersed in acetone and trapped on holey carbon membranes. The particle size measurements were carried out using the HELOS Rados laser diffraction to give the average size distribution (Sympatec Gmbh, Clausthal-Zellerfeld, Germany). For the sample preparation, 5 mg of samples are mixed with 0.1 M sodium pyrophosphate aqueous solution to give a homogeneous suspension. The suspension was later dispersed with an ultrasonic probe at 70 W for 10 min using distilled water before analysis. Fourier-transform infrared (FTIR) spectrometer analysis was carried out on disc-pallet sample, scanned at a resolution of 4 cm^−1^ over a wavenumber region of 450 to 4000 cm^−1^ using the FTIR spectrometer (Spectrum RX1 FTIR system, Perkin Elmer, Waltham, M.A., USA). To prepare the disc-pallet, two milligram of sample were mixed with 80 mg of potassium bromide (KBr, Aldrich, Germany) and ground into a fine powder, before compressing into a disc-pallet. The characteristic peaks of IR transmission spectra were recorded in triplicates for each sample and the results were averaged. The thermal analysis was analysis carried out using a Mettler Toledo thermal analyzer (Columbus, O.H., USA), with a heating rate of 10 °C min^−1^ from room temperature to 1200 °C under a nitrogen atmosphere. For solid samples, the UV–Vis diffuse reflectance spectra (DR UV–Vis) were measured by a Perkin Elmer Lambda 900 ultraviolet-visible/near-infrared (UV–Vis/NIR, Waltham, M.A., USA) using a polytetrafluoroethylene polymer as a standard background, scanning in the wavelength ranges from 250 to 900 nm against absorbance. For liquid samples, the UV–Vis spectra were measured using a Thermo Scientific GENESYS 10S (Madison, W.I., USA) from 500 to 800 nm. The X-ray photoelectron spectroscopy (XPS, JEOL JPS-9200) spectra (JEOL, Tokyo, Japan) were recorded using a standard Mg-Kα radiation as the X-ray source. A charge neutralizer was used to reduce charging effects, and data were taken using a pass energy of 10 eV. All the binding energies are corrected based on the adventitious C 1s peak (284.8 eV) with Shirley background was used in the peak fitting. For XPS peaks processing, deconvolution was performed using Gaussian (70%)-Lorentzian (30%). The fluorescence spectra of samples were measured on a photoluminescence (PL) spectroscopy (JASCO FP-8500, Tokyo, Japan) with both of excitation and emission bandwidths were fixed at 5 nm.

### 2.3. Catalytic Activity 

In this study, we have investigated the catalytic performance of PS-SnS_2-x_ for wastewater treatment by considering the degradation of MB dye in aqueous solution under sunlight irradiation at room temperature. Notably, MB with aromatic structures (Figure S1) is the most common basic/cationic dyeing materials for silk and cotton, in the textiles industries and chosen as the source for water pollutants [21]. Human consumption on water-polluted dye from MB had caused health problems such as digestive and respiratory systems failure, nausea, and vomiting as well as profuse sweating [22,23,24]. Firstly, the suspension that consists of 2.0 mL of 0.1 mM MB added to 50 mg of the PS-SnS_2-x_ in a quartz cuvette, stirred in the dark for 180 min to ensure the adsorption-desorption equilibrium. After 180 min, the absorption spectra were recorded through time-dependent UV–Vis at 30 min intervals over the scanning wavelength from 200 to 600 nm, at room temperature. The MB degradation was monitored from decreasing UV–Vis intensity at the absorption band of 638 nm.

## 3. Results and Discussion

### 3.1. Characterization of Photochemical Synthesis of Tin Disufides (PS-SnS_2-x_)

**Figure 1:**
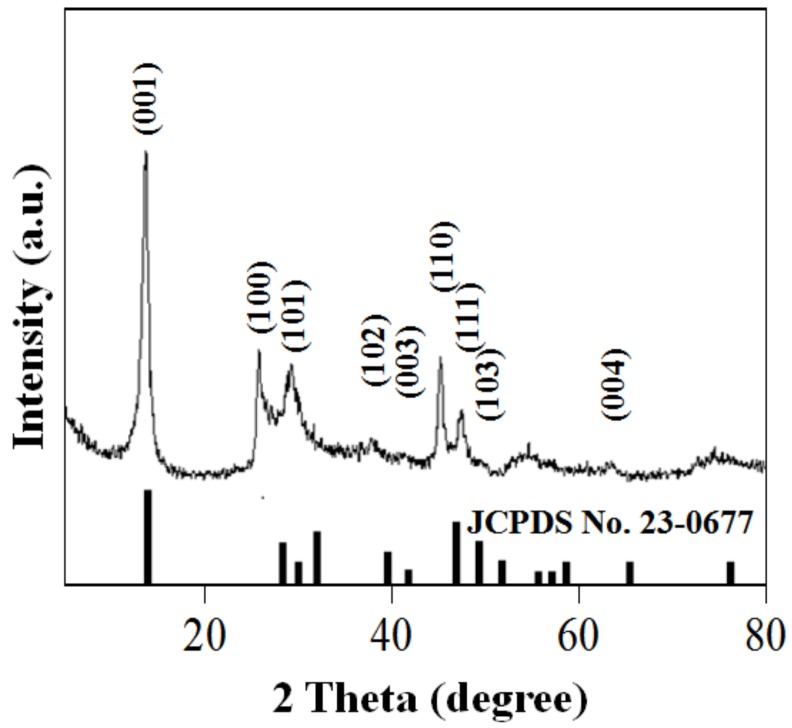
X-ray diffraction (XRD) pattern of the PS-SnS_2-x_, indexed to the hexagonal structure of tin di/sulfide JCPDS card No. 23-0677 (vertical lines).

The phase composition and crystal structure of PS-SnS_2-x_ were characterized using XRD as shown in Figure 1. As can be seen, the XRD peak positions are in good agreement with the standard diffraction data of JCPDS no. 23-0677, representing a hexagonally closed-packed arrangement of SnS_2-x_ with the space group of P-3m1. Clearly, intense peaks of SnS_2-x_ are measured at 15.1°, 28.4°, 32.3°, and 50.1° corresponding to crystal (0 0 1) plan, (1 0 0) plan, (1 0 1) plan and (1 1 0) plan, respectively. By analyzing Bragg reflection measured at 2θ = 15.1°, the favored orientation growth of PS-SnS_2-x_ is parallel to the C-direction, indicating a preferential stacking along the (0 0 1) direction stronger than the (1 0 0) and (1 0 1) planes due to the anisotropic arrangements [25]. From the XRD analysis, the XRD diffractogram match well with the assignment of a 2T-type hexagonal layered for nanosheets PS-SnS_2-x_ having well-ordered structure due to weak van der Waals forces [26,27,28,29]. The *d*-spacing (2θ = 15.1°) of nanosheets PS-SnS_2-x_ estimated at 5.8626 Å by using the following Bragg’s Law:

2*d* sin θ = nλ (1)

where n is a positive integer and λ is the wavelength of the incident wave. Furthermore, the lattice constants of cell parameters were determined at a= b= 3.589 ± 0.001 Å and c= 5.862 ± 0.004 Å, with c/a ratio and atomic packing factor of 1.633 and 0.74, respectively, as calculated using the following Bravais Lattice equation:

(2) $$\frac{1}{d_{\mathrm{hkl}}^{2}}=\frac{4}{3}\left(\frac{h^{2}+\mathrm{hk}+k^{2}}{a^{2}}\right)+\frac{l^{2}}{c^{2}}$$

where *d* is the distance of adjacent planes (h k l), a and c are the lattice parameters of the hexagonal structure. Notably, the as-obtained value for c corresponding to integral numbers of the inter-lamellar spacing is close to d-spacing obtained by Bragg’s Law and literature values (5.899 Å) [25,28]. From the XRD pattern, the crystalline PS-SnS_2-x_ was free from any contamination by other phases such as tin (II) oxide (SnO), or tin (IV) oxide (SnO_2_). Judging from the intense XRD pattern, it was evident that the PS-SnS_2-x_ was having good crystallinity and anisotropically well-developed at (0 0 1) planes that stacked along C-axis without susceptible faults separation in the nanosheet arrangements [29]. 

For 2D nanostructured materials, the planes thickness along C-axis can be estimated from the half-width XRD peaks along the oriented axis using the following the Debye–Scherrer equation [29,30]:

*d* = kλ/(β × cos θ) (3)

where θ is the Bragg diffraction angle, β is the full width at the half-height maxima (FWHM) of the desired XRD peaks, k is the Scherrer constant of 0.9 for typical crystallites, λ is the X-ray wavelength of Cu Kα = 0.15406 nm, and *d* is the particle size of the crystal. By using the equation, the nanosheet thickness of PS-SnS_2-x_ was estimated to be 26 nm according to the (0 0 1) peak. It is of interest, the SnS_2-x_ samples on different irradiation at 30 and 60 W were also scanned using XRD. In this case, based on XRD pattern in Figure S2, only the (0 0 1) plane are reasonably measured, while the (1 0 0) and (1 0 1) planes are non-distinguished peaks. Based on kinetic feasibility, the photochemical synthesis of PS-SnS_2-x_ had promoted the metal species nucleation, aggregation growth, and self-assembly preferentially along (0 0 1) plane due to solvents and precursors characteristic [31]. 

**Figure 2:**
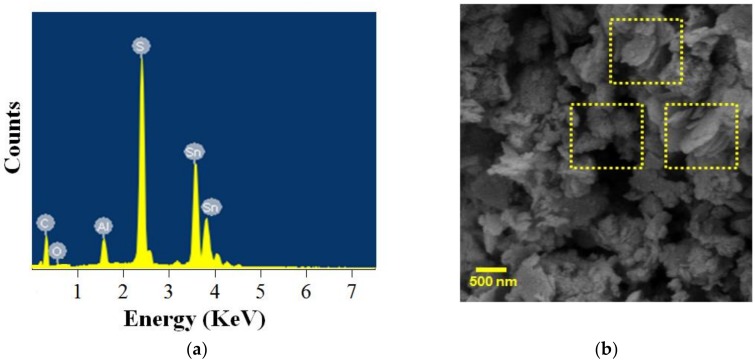
(**a**) Energy dispersive X-ray spectroscopy (EDX) analysis spectra on selected-area electron diffraction (SAED) of PS-SnS_2-x_. (**b**) Field emission scanning electron microscopy (FESEM) micrograph of the PS-SnS_2-x_ at 30,000× magnification (the dotted box suggesting a layered sheet-like structure).

To confirm the sample composition and purity, the PS-SnS_2-x_ was analyzed by the energy dispersive X-ray analysis (EDX) as shown in Figure 2a. The EDX spectrum of the PS-SnS_2-x_ shows the elemental composition of sulfur (S) at 2.0 to 2.4 keV, and tin (Sn) at 3.5 to 3.8 keV, with considerable aluminum (Al), detected due to sample preparations. The presence of carbon (C) and oxygen (O) may have been caused by experimental conditions and solution applied [32]. Table 1 represents the EDX depth analysis with the elemental composition for the PS-SnS_2-x_ was confirmed to be S (at 17.09%) and Sn (at 32.31%), without any elemental impurities being detected. EDX analysis showed the atomic ratio between Sn and S was 1:1.9 (error <0.05%), which is very close to the theoretical stoichiometry of SnS_2-x_, thus, confirming its chemical composition as SnS_2-x_. In considering the experimental results, the photochemical synthesis produces SnS_2-x_ based on the off-stoichiometry findings. This finding indicated that the highly purified SnS_2-x_ was successfully prepared from the photochemical synthesis and corroborated the XRD analysis. As shown in Figure 2b, the PS-SnS_2-x_ was distributed as layered materials consist of a high agglomeration of sheet-like aggregates with an average size distribution around 800 nm. Predominantly, the PS-SnS_2-x_ has similar morphology to that of other layered metal di/sulfides, replicating agglomerated sheet-like materials morphology [33]. The presence of agglomeration is conceived to be due to capping effects by additives during termination of S at (0 0 1) facets on crystals ripening process [34,35]. Furthermore, our results share the common observation of nanosheets with previous findings [31,36]. 

**Figure 3:**
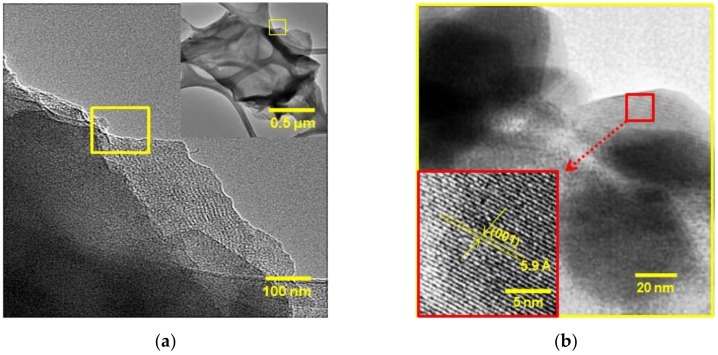
TEM micrograph of (**a**) photochemical synthesis of tin di/sulfides (PS-SnS_2-x_), inset: corresponding PS-SnS_2-x_ at lower magnification, and (**b**) lattice fringes at the (0 0 1) plane having lamellar structures with an inter-planar distance of 5.9 Å.

Transmission electron microscopy (TEM) was used to assess the inter-spacing structures over a few-layer of nanosheet PS-SnS_2-x_ as shown in Figure 3. In Figure 3, TEM images at different magnification on PS-SnS_2-x_ further confirmed the surface nanosheet shape with (0 0 1) facet, and in good agreement with the XRD and FESEM analysis. TEM images in Figure 3a show the sheet-like morphology is similar to those observed for 2D layered materials such as graphene [37,38]. Also, we can see that aggregates with a size of more than 500 nm are composed of a large number of nanosheets (inset Figure 3a). TEM images in Figure 3b revealed lamellar structure from thin strips of regular lattice fringes with d-spacing of 0.59 nm, which is consistent with the inter-planar distance of (0 0 1) planes in hexagonal SnS_2-x_ crystal [33,39]. It is consistent with the above XRD and FESEM analysis results that the PS-SnS_2-x_ is highly crystallized aggregates. From the XRD, SEM and TEM analysis, it can, therefore, be deduced that layered structures are interlinked over multiple nanosheets of hexagonal SnS_2-x_ crystals [40,41] to give highly agglomeration of sheet-like aggregates. 

**Figure 4:**
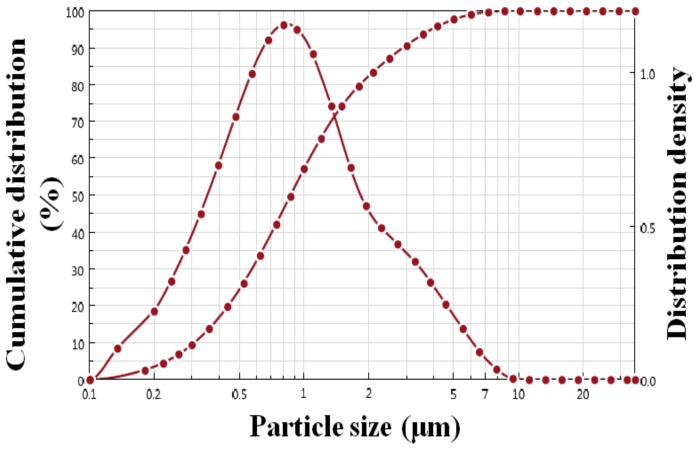
Particle size distribution of PS-SnS_2-x_ obtained by laser diffraction on the volume-specific surface of particles.

To quantify the particle size of PS-SnS_2-x_, Figure 4 shows the particle size distribution of PS-SnS_2-x_ by laser diffraction on the volume-specific surface of particles. As can be seen, the measured average particle size was found to be 870 nm, showing almost a binomial distribution of 0.31–2.91 μm. The density distribution curve is monomodal with a high amount centered in the mean region. This finding is in good agreement with FESEM and TEM morphology analysis. The measured particle size for PS-SnS_2-x_ at 870 nm is most likely due to strongly bound S-Sn-S stacked units. It is worth noting that sheet-like PS-SnS_2-x_ consists of Sn cations with two layers of hexagonal closed-packed S anions and adjacent S–Sn–S sandwich order held together by van der Waals interactions [25]. The S atoms occupy this kind of sandwich order to give a molecular layer of saturated close packing to the Sn atoms [26]. Compared with other synthetic SnS_2-x_, the sheet-like particle size obtained from the photochemical synthesis was higher than that works presented by Taleblou et al. (685 nm) [32] and Fu et al. (275 nm) [36]. To date, owing to the large particles size and sheet-like arrangement exhibiting superior electronic properties, SnS_2-x_ were developed as photodetectors, semiconductors, supercapacitors, and batteries [28,31,33,42]. 

**Figure 5:**
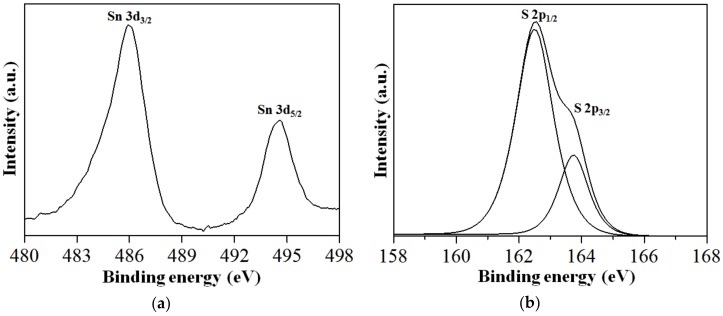
XPS profile of the PS-SnS_2-x_: (**a**) Sn 3d core levels and (**b**) S 2p core levels.

The XPS study was conducted to understand the surface electronic states and binding feature between the PS-SnS_2-x_ as shown in Figure 5. Briefly, the XPS spectrum (Figure 5) provides a complete view on the surface elemental evolution of the Sn 3d and S 2p core level of the PS-SnS_2-x_ nanostructures. In Figure 5a, the two strong deconvoluted peaks at 486.3 and 494.7 eV with a spin-orbit doublet splitting of 8.4 eV, can be assigned to Sn 3d_3/2_ and Sn 3d_5/2_, respectively. These peaks match closely to the characteristic Sn^4+^ peaks in SnS_2-x_ [27,43,44]. At a higher resolution, XPS spectra (Figure 5b) of S 2p displayed a pair of doublets spin-orbital peaks centered at 162.5 and 163.7 eV, which were attributed to S 2p_1/2_ and S 2p_3/2_, respectively. The S 2p core level region displayed a spin-orbit doublet splitting of 1.2 eV corresponding to S^2−^ species that are common to SnS_2-x_ phases. Altogether, the XPS profiles on binding energies of Sn^4+^ and S^2−^ further confirmed the presence of Sn and S in the PS-SnS_2-x_ and is in good agreement with the literature [25,30,45]. 

**Figure 6:**
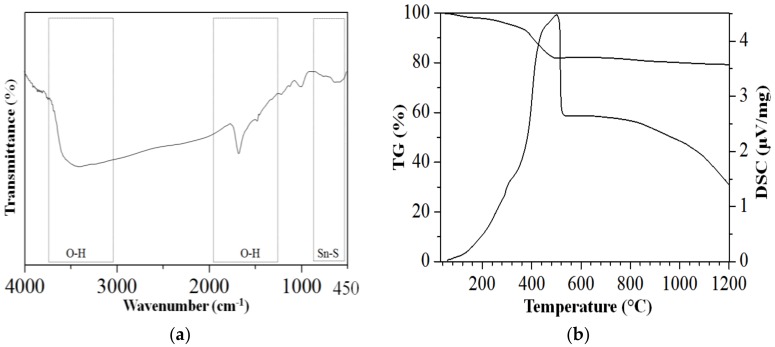
Structural analysis of the PS-SnS_2-x_: (**a**) Fourier transform infrared (FTIR) spectra and (**b**) Thermal-differential scanning calorimetry thermogram.

To give further insight, the chemical structure and compound content of PS-SnS_2-x_ was examined using FTIR and thermal analysis as shown in Figure 6. Based on FTIR analysis (Figure 6a), very intense broad bands at 3400 and 1633 cm^−1^ can be assigned to hydrogen-bonded hydroxyl groups (O–H) vibration indicating strong water bounds by intermolecular hydrogen bonding in the structure. Since sheet-like materials are highly hydrophilic and easily trapped moisture, the broad band in Figure 6a, is very much associated with the presence of water consistent in complete agreement with the literatures [32,34]. More importantly, the presence of the vibrational band at 628 cm^−1^ is attributed to the Sn-S bond. According to the thermal analysis, in Figure 6b, the remained phase after 450 °C is corresponding to the residual inorganic contents with the final weight loss of 19.1%. Based on the thermogram, the initial weight of 6% at less than 200 °C (in Figure 6b) are attributed to the adsorbed water moisture within the sheet-like structure as proposed by FTIR analysis (in Figure 6a). The final weight loss of an additional 13.1% after 250 °C are ascribed to the removal of the adsorbed oxygen fixed within the nanosheet arrangements [35,44]. The final residual mass after heat treatment in the air was found to be 81.9% at analysis temperature up to 1200 °C, strongly suggest PS-SnS_2-x_ is thermally stable.

**Figure 7:**
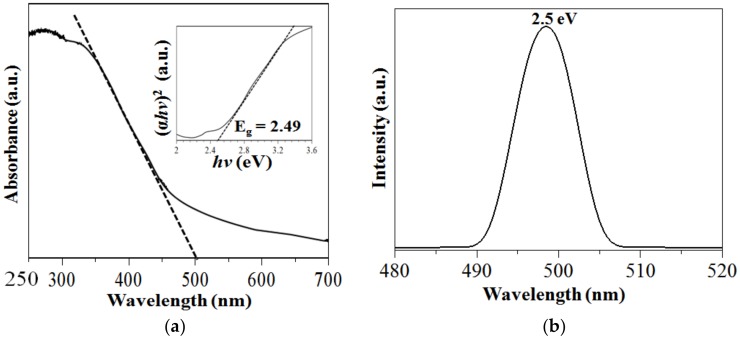
Optical analysis for the PS-SnS_2-x_ (**a**) UV–Visible diffuse reflectance spectrum (DR UV–Vis) with inset Tauc plot, and (**b**) Photoluminescence spectra from λ excitation at 280 nm.

The optical property of PS-SnS_2-x_ was investigated using DR UV–Vis and PL as shown in Figure 7. According to Figure 7a, the PS-SnS_2-x_ gives broad photo-absorption bands across the ultraviolet and visible wavelengths from 280 to 500 nm. From the DR UV–Vis absorption, the optical bandgap (Eg) value of the PS-SnS_2-x_ was determined using a Tauc plot based on the following formula:

αhυ = A(hυ − E_g_)^2^ (4)

where α is the absorption coefficient, hυ is the photon energy, and A is constant. From the inset Figure 7b, by extrapolating the linear part of the transformed (αhν)^2^ against photon energy plots the indirect gaps of PS-SnS_2-x_ were calculated at 2.49 eV. This value is in good agreement with the previous reports on nanostructured SnS_2-x_ (2.18–2.50 eV) [46,47]. Moreover, the theoretical optical bandgap energy of the PS-SnS_2-x_ was calculated using the following Einstein’s photoelectric effect formula [36,48]:

E_g_ = (1240/λ) eV (5)

where λ is the lower cut-off wavelength in nm. From the onset of the absorption edge, at 500 nm the Eg value is estimated to be 2.48 eV [36]. The broad spectrum measured due to the intrinsic bandgap transition implies that the prepared PS-SnS_2-x_ are useful as sunlight response photocatalysts for degradation of organic pollutants. To further confirm the E_g_ value, the PS-SnS_2-x_ was analyzed via the PL technique. In Figure 7b, the PL spectra show a strong excitation peak at 496 nm, indicating charge migration from recombination of excitons of the conduction band to the valence band [36,40]. From the strong PL peak, the E_g_ was estimated directly at 2.5 eV by using aforementioned formula (5) [36] and confirmed with the value calculated from DR UV–Vis analysis. 

### 3.2. Photocatalytic Activity on Sunlight Response

**Figure 8:**
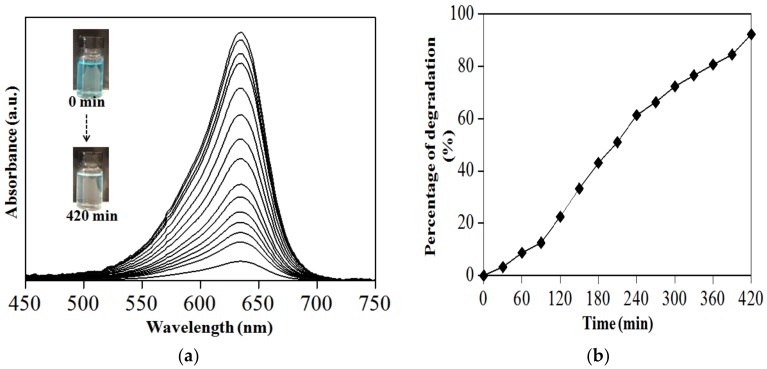
Degradation of methylene blue (MB) for 420 min (**a**) PL changes under sunlight response and (**b**) plot of degradation (%) against time.

As highlighted earlier, the challenge in the textiles industries is the dye-waste treatments having to diminish their contaminant impact on environments, in the end, providing reusable clean water by degradation of the dye pollutant. To demonstrate the photocatalytic activity for this study, the PS-SnS_2-x_ underwent a catalytic performance evaluation under sunlight on the degradation of MB dye as the source of water pollutants. Once the dye solution and PS-SnS_2-x_ mixtures reached adsorption−desorption equilibrium (Figure S3), the solution was then directly monitored for a photo-degradation reaction. The UV−Vis absorption spectra of MB and their percentage of degradation as a function of reaction time under sunlight irradiation at room temperature are presented in Figure 8. In this study, the percentage of degradation for MB was calculated using the following equation:

Percentage of degradation (%) = [(C_o_ − C*_t_*)/(C_o_)] × 100% (6)

where C_o_ is the initial concentration and C*_t_* is the concentration at time *t*. Based on Figure 8b, MB directly degrades at 3% after 30 min continuous mixing with PS-SnS_2-x_. From the photo-degradation evaluation, MB further degraded to 51% and 72%, at 210 and 300 min, respectively. Overall, the degradation processes for MB in the presence of PS-SnS_2-x_ under sunlight give a total degradation of 92% after 420 min. To compare, MB did not degrade when exposed to sunlight without the PS-SnS_2-x_ in the control experiments setting. Nonetheless, the agglomerated sheet-like morphology of PS-SnS_2-x_ may result in fast photo-generated charge transfer/separation dynamics and significantly contributed to the photocatalytic capability. On the other hand, the UV–Vis absorption spectra did not give any significant change in dark conditions, indicating that the photo-degradation process is not physical adsorption but follow photocatalytic mechanism from sunlight response. Intriguingly, the photo-degradation process using PS-SnS_2-x_ did not need of any prevalent oxidizing agents such as H_2_O_2_ and KMnO_4_ or reducing agents such as NaBH_4_ and LiAlH_4_, yet the performance is comparable to that of the previously reported SnS_2-x_ as a catalyst [49,50,51,52]. Moreover, the PS-SnS_2-x_ shows photo-degradation on MB in room temperature without any pH adjustment indicating suitability for wastewater treatments.

**Figure 9:**
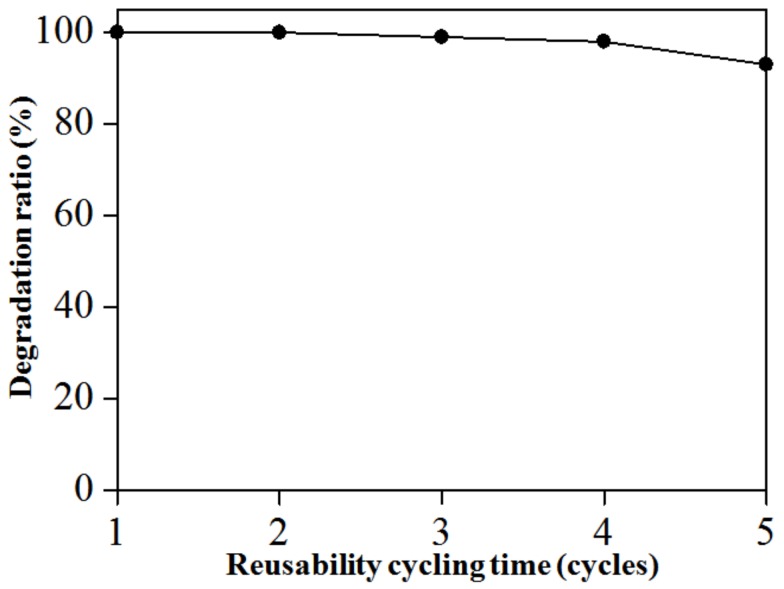
Degradation evolution profile of PS-SnS_2-x_ under sunlight response on MB for the first five cycles.

To study the reaction kinetics, the photo-degradation of MB using PS-SnS_2-x_ gives both an exponential and linear relationship of pseudo-first-order kinetics as shown in Figure S4. From the following kinetics equation:

ln (C/C_0_) = kt (7)

which, C_0_ is the initial concentration and C*_t_* is the concentration after a certain time (t) on corresponding MB, the value of rate constant, k was calculated at 3.32 × 10^−3^ min^−1^ and half-life, t_1/2_ at 209 min. In comparison, the k has similar value as in reported data (3.2−4.8 × 10^−3^ min^−1^) for photo-degradation of dye pollutants using SnS_2-x_ nanoparticles [17,25,53,54,55,56]. Furthermore, in Figure 9, the degradation evolution profile of PS-SnS_2-x_ on photo-degradation of MB is represented from the plot of degradation ratio against reusability cycling time. From the graph, the PS-SnS_2-x_ show stable reusability for up to five cycle usages on the photo-degradation of MB. In details, the PS-SnS_2-x_ show 100% degradation ratio of MB pollutants for first and second cycles, before slightly decrease to 99% and 98% during the third and fourth cycles, respectively. In the fifth cycles, the reusability of PS-SnS_2-x_ is maintained at 93%, suggesting good photo-degradation reusability on MB under sunlight response as compared to similar SnS_2-x_-type photocatalyst and vital for its industrial application prospects [39]. 

### 3.3. Plausible Mechanisme 

**Figure 10:**
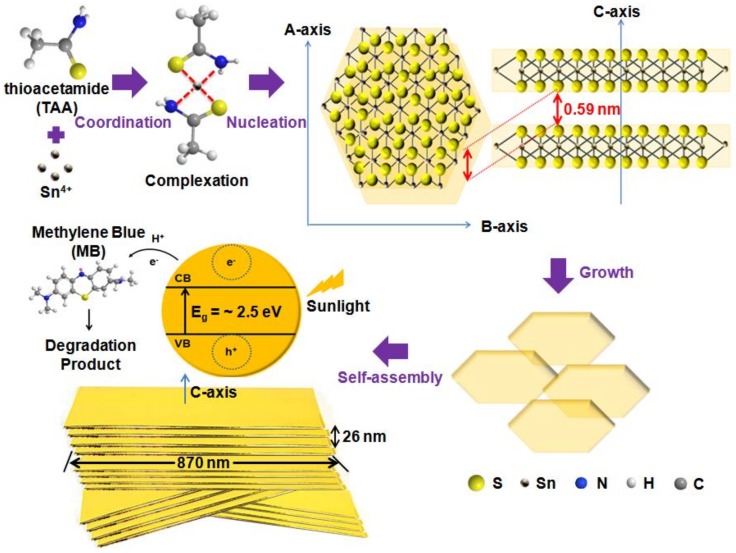
Schematic formation of PS-SnS_2-x_ with sunlight response.

On the bases of the above experimental results and discussion, a plausible growth mechanism of the nanosheet PS-SnS_2-x_ can be illustrated as shown in Figure 10. In view of the chemical environment and crystal growth, the formation of the 2D nanosheet PS-SnS_2-x_ is mainly as a result of the adsorption on chemical precursors of TAA and SnCl_4_.5H_2_O in the aqueous solvents on the (0 0 1) facet. During the initial stage of crystal nucleation and growth processes in the photochemical synthesis, the SnCl_4_ interact to form a complex with TAA under acidic condition. In this approach, TAA was chosen as the sulfur (S) source permitting only slow decomposition rate in aqueous solution to give a lower degree of supersaturation [25,35,49]. The overall photochemical synthesis for the H_2_S formation on halogen-lamp irradiation is represented in the following reactions:

CH_3_CSNH_2_ + H_2_O → CH_3_CONH_2_ + H_2_S (8)

H_2_S → 2H^+^ + S^2−^ (9)

(10) $${\mathrm{Sn}}^{4+}+S^{2-}\overset{\mathrm{hv}}{\to }{\mathrm{SnS}}_{2-x}$$

At the early stage in reaction 8, the hydrolysis of TAA releases H_2_S under acidic medium. Based on the above reactions (Equations (9) and (10)), each TAA molecule is supposed to coordinate with at least one Sn^4+^ ions through its N atoms in the TAA/SnCl_4_ solution to from Sn--CH_3_CONH_2_, a Sn (IV)-type complexes. To give the sandwich layered structures, the reaction continues by the formation of inter-twisted H-bonds formed between H atoms and N atoms of two nearby CH_3_CONH_2_ molecules. The 2D inter-digitations in each layer consist of similar S atoms arrayed in each Sn–CH_3_CONH_2_, subsequently giving the uniform orientation of S-Sn-S with each layer containing two S-atom and one Sn-atom of opposite charge. Under halogen-lamp irradiation in Equation (9), the sandwich layered of Sn (IV) complexes starts to degrade to produce Sn^4+^ ions at the high reaction temperature (>100 °C). In an instant, the strong coordination bonds between the H_2_S and Sn^4+^ were weakening the S–H bond further broke to release S^2−^ ions, and combine with Sn^4+^ ions to initiate the SnS_2-x_ nucleation. After a certain period, the readily nucleated SnS_2-x_ tend to form matured SnS_2-x_ particles and grow larger by recrystallization of growth processes (Equation (10)). In due course, the nanosheet PS-SnS_2-x_ self-assembled through minimizing the surface energy by crystal suppression and Ostwald ripening processes. The Ostwald ripening processes that stimulate anisotropic growth are most probably responsible for sheet-like morphology for PS-SnS_2-x_ [34,49,54]. As validated by the morphology and crystal structure analysis, the nanosheets became thicker and larger as the ripening progress obeyed the distinct nature of hexagonal berndtite SnS_2-x_, to take the form of most stable nanosheets stacked by at least triple layers of S–Sn–S [57,58,59]. Notably, incomplete reduction by H_2_S might contribute to the considerable formation of Sn-S. To highlight the photocatalytic behavior, SnS_2-x_ possess a mixed character for electronic bands from metal d-orbitals and p-orbitals of the chalcogen species, which is crucial in semiconductor photocatalytic application. Since SnS_2_ is an n-type semiconductor, the photocatalytic degradation of dye pollutant is corresponding to the reduction process. In principle, after PS-SnS_2-x_ absorbed photon energy from sunlight, the valence band (VB) electrons (e−) from two negative charge carriers of are excited to the conduction band (CB) and create holes (h+) carriers from four positive charges of Sn [27,59,60,61]. For PS-SnS_2-x_, the large sheet-like structures and thin thickness are suitable active sites for optimum electrons and holes migration to reaction sites of the surface for reduction of MB dye. For the sunlight response of PS-SnS_2-x_, its might be contributed by the highly dispersive conduction band of Sn central metal ion with a d^10^ electronic configuration [62,63,64,65]. In this context, the sunlight stimulates photoexcited CB where the separated e− act to reduce the MB dye [16,18,49]. In the same manner reported previously [16,17,18,60,61], the proposed photo-degradation of MB under sunlight response in the presence of PS-SnS_2-x_ in aqueous solution that obeyed a direct reduction mechanism is as follows:

(11) $${\mathrm{SnS}}_{2^{-}}\overset{\mathrm{hv}}{\to }{\mathrm{SnS}}_{2}(e^{-}+h^{+})$$

(12) $$H_{2}O+2H^{+}\to \frac{1}{2}O_{2}+2H^{+}$$

(13) $$C_{16}H_{18}N_{3}S^{2+}+\frac{15}{2}O_{2}\to 16{\mathrm{CO}}_{2}+3{{\mathrm{NO}}_{3}}^{-}+{\mathrm{SO}}_{4}{}^{2-}+6H^{+}+6H_{2}O$$

The final products for photo-degradation of MB give carbon dioxide, sulfate, nitrate, and ammonium ions from carbon, sulfur and nitrogen heteroatoms [60,61].

## 4. Conclusions

To summarize, we have developed a facile preparation from the photochemical synthesis of nanosheet PS-SnS_2-x_ using TAA as the S source and Sn salts precursor in an aqueous system. The XRD analysis indicated that the PS-SnS_2-x_ form hexagonally closed-packed crystals. The TEM, FESEM, and EDX analyses showed sheet-like aggregates with good elemental distributions in the PS-SnS_2-x_. The particle size analysis suggested that the average size for PS-SnS_2-x_ is 870 nm. XPS analysis confirmed that Sn^4+^ and S^2−^ are present in the nanosheet aggregates. PL and DR UV–Vis spectra suggested that the samples are capable of absorbing light with corresponding E_g_ at ~2.5 eV. We explored the catalytic activities via MB photo-degradation under sunlight in the neutral water medium, without the presence of harsh oxidizing or reducing agents. The kinetics reaction of photo-degradation on MB was investigated to follow pseudo-first-order kinetics model (k = 3.32 × 10^−3^ min^−1^) with good reusability (up to five cycles). In the future, the photochemical synthesis presented here is expected to offer a feasible procedure on the green protocol for the preparation of 2D nanosheets of semiconductor materials in sustainable development for water purification and solar water splitting applications. 

## Figures and Tables

**Table 1:** EDX depth analysis for photochemical synthesis of tin di/sulfide (PS-SnS_2-x_) under aluminum coating.

Elements	Atomic Percentage (%)
Carbon (C)	39.26
Aluminum (Al)	6.15
Oxygen (O)	5.28
Sulfur (S)	32.31
Tin (Sn)	17.09

## Supplementary Materials

The following are available online at http://www.mdpi.com/2079-4991/9/2/264/s1, Figure S1: Methylene blue (MB) structures with chemical formula of C_16_H_18_N_3_S without the presence of Cl- to simplify the structures, Figure S2: XRD patterns for tin di/sulfides (SnS_2-x_) prepared on different irradiation at (a) 30 W and (b) 60 W, Figure S3: Adsorption-desorption equilibrium in dark condition of PS-SnS_2-x_ on MB for 180 min. Figure S4: Plot on concentration of MB as a function of irradiation time for 180 min.

## References

[1] Oelgemöller M., Jung C., Mattay J. (2007). Green Photochemistry: Production of Fine Chemicals with Sunlight. Pure Appl. Chem..

[2] Matmin J., Affendi I., Endud S. (2018). Direct-Continuous Preparation of Nanostructured Titania-Silica Using Surfactant-Free Non-Scaffold Rice Starch Template. Nanomaterials.

[3] Matmin J., Affendi I., Ibrahim S.I., Endud S. (2018). Additive-Free Rice Starch-Assisted Synthesis of Spherical Nanostructured Hematite for Degradation of Dye Contaminant. Nanomaterials.

[4] Yoon T.P., Ischay M.A., Du J. (2010). Visible Light Photocatalysis as a Greener Approach to Photochemical Synthesis. Nat. Chem..

[5] Gu H., Yang Y., Tian J., Shi G. (2013). Photochemical Synthesis of Noble Metal (Ag, Pd, Au, Pt) on Graphene/ZnO Multihybrid Nanoarchitectures as Electrocatalysis for H_2_O_2_ Reduction. ACS Appl. Mater. Interfaces..

[6] Wei H., Huang K., Wang D., Zhang R., Ge B., Ma J., Wen B., Zhang S., Li Q., Lei M., Zhang C. (2017). Iced Photochemical Reduction to Synthesize Atomically Dispersed Metals by Suppressing Nanocrystal Growth. Nat. Commun..

[7] Watanabe K., Menzel D., Nilius N., Freund H.J. (2006). Photochemistry on Metal Nanoparticles. Chem. Rev..

[8] Fang Z., Fan Y., Liu Y. (2011). Photochemical Synthesis and Photocatalysis Application of ZnS/Amorphous Carbon Nanotubes Composites. Front. Optoelectron. China.

[9] Placido T., Comparelli R., Giannici F., Cozzoli P.D., Capitani G., Striccoli M., Agostiano A., Curri M.L. (2009). Photochemical Synthesis of Water-Soluble Gold Nanorods: the Role of Silver in Assisting Anisotropic Growth. Chem. Mater..

[10] Zhao W.B., Zhu J.J., Chen H.Y. (2003). Photochemical Synthesis of Au and Ag Nanowires on a Porous Aluminum Oxide Template. J. Cryst. Growth..

[11] Chen Y., Fan Z., Zhang Z., Niu W., Li C., Yang N., Chen B., Zhang H. (2018). Two-Dimensional Metal Nanomaterials: Synthesis, Properties, and Applications. Chem. Rev..

[12] Huang X., Qi X., Huang Y., Li S., Xue C., Gan C.L., Boey F., Zhang H. (2010). Photochemically Controlled Synthesis of Anisotropic Au Nanostructures: Platelet-Like Au Nanorods and Six-Star Au Nanoparticles. ACS Nano..

[13] Bharath G., Anwer S., Mangalaraja R.V., Alhseinat E., Banat F., Ponpandian N. (2018). Sunlight-Induced Photochemical Synthesis of Au Nanodots on α-Fe_2_O_3_@ Reduced Graphene Oxide Nanocomposite and their Enhanced Heterogeneous Catalytic Properties. Sci. Rep..

[14] Chen C., Zhou P., Wang N., Ma Y., San H. (2018). UV-Assisted Photochemical Synthesis of Reduced Graphene Oxide/ZnO Nanowires Composite for Photoresponse Enhancement in UV Photodetectors. Nanomaterials.

[15] Pu F., Ran X., Guan M., Huang Y., Ren J., Qu X. (2018). Biomolecule-Templated Photochemical Synthesis of Silver Nanoparticles: Multiple Readouts of Localized Surface Plasmon Resonance for Pattern Recognition. Nano Res..

[16] Soltani N., Saion E., Hussein M.Z., Erfani M., Abedini A., Bahmanrokh G., Navasery M., Vaziri P. (2012). Visible light-induced degradation of methylene blue in the presence of photocatalytic ZnS and CdS nanoparticles. Int. J. Mol. Sci..

[17] Balu S., Uma K., Pan G.T., Yang T., Ramaraj S. (2018). Degradation of Methylene Blue Dye in the Presence of Visible Light using SiO_2_ @ α-Fe_2_O_3_ Nanocomposites Deposited on SnS_2_ flowers. Materials.

[18] Dhanasekar M., Ratha S., Rout C.S., Bhat S.V. (2017). Efficient Sono-Photocatalytic Degradation of Methylene Blue Using Nickel Molybdate Nanosheets under Diffused Sunlight. J. Environ. Chem. Eng..

[19] Oelgemoller M. (2016). Solar Photochemical Synthesis: From the Beginnings of Organic Photochemistry to the Solar Manufacturing of Commodity Chemicals. Chem. Rev..

[20] Oelgemöller M., Healy N., de Oliveira L., Jung C., Mattay J. (2006). Green Photochemistry: Solar-Chemical Synthesis of Juglone with Medium Concentrated Sunlight. Green Chem..

[21] Stichnoth D., Kölle P., Kimbrough T.J., Riedle E., de Vivie-Riedle R., Trauner D. (2014). Photochemical Formation of Intricarene. Nat. Commun..

[22] Aikins G.A., Fay A.C. (1932). Effect of Light on the Reduction of Methylene Blue in Milk. J. Agric. Res..

[23] Djellabi R., Ghorab M.F., Sehili T. (2017). Simultaneous Removal of Methylene Blue and Hexavalent Chromium from Water using TiO_2_/Fe (III)/H_2_O_2_/Sunlight. CLEAN Soil Air Water.

[24] Molla A., Sahu M., Hussain S. (2015). Under Dark and Visible Light: Fast Degradation of Methylene Blue in the Presence of Ag–In–Ni–S Nanocomposites. J. Mater. Chem. A..

[25] Park S., Park J., Selvaraj R., Kim Y. (2015). Facile Microwave-Assisted Synthesis of SnS_2_ Nanoparticles for Visible-Light Responsive Photocatalyst. J. Ind. Eng. Chem..

[26] Dubrovskii G.B. (1998). Crystal Structure and Electronic Spectrum of SnS_2_. Phys. Solid State..

[27] Yu J., Xu C.Y., Ma F.X., Hu S.P., Zhang Y.W., Zhen L. (2014). Monodisperse SnS_2_ Nanosheets for High-Performance Photocatalytic Hydrogen Generation. ACS Appl. Mater. Interfaces..

[28] Huang Y., Sutter E., Sadowski J.T., Cotlet M., Monti O.L., Racke D.A., Neupane M.R., Wickramaratne D., Lake R.K., Parkinson B.A., Sutter P. (2014). Tin Disulfide—An Emerging Layered Metal Dichalcogenide Semiconductor: Materials Properties and Device Characteristics. ACS Nano..

[29] Zhang Z., Zhao H., Du Z., Chang X., Zhao L., Du X., Li Z., Teng Y., Fang J., Świerczek K. (2017). (101) Plane-Oriented SnS_2_ Nanoplates with Carbon Coating: a High-Rate and Cycle-Stable Anode Material for Lithium Ion Batteries. ACS Appl. Mater. Interfaces..

[30] Wei R., Hu J., Zhou T., Zhou X., Liu J., Li J. (2014). Ultrathin SnS_2_ Nanosheets with Exposed {0 0 1} Facets and Enhanced Photocatalytic Properties. Acta Mater..

[31] Parveen N., Ansari S.A., Alamri H.R., Ansari M.O., Khan Z., Cho M.H. (2018). Facile Synthesis of SnS_2_ Nanostructures with Different Morphologies for High-Performance Supercapacitor Applications. ACS Omega.

[32] Taleblou M., Borhani E., Yarmand B., Kolahi A.R. (2018). Structural and Optoelectrical Properties of Single Phase SnS_2_ Thin Films at Various Substrate Temperatures by Spray Pyrolysis. Iran. J. Mater..

[33] Liu S., Lu X., Xie J., Cao G., Zhu T., Zhao X. (2013). Preferential C-Axis Orientation of Ultrathin SnS_2_ Nanoplates on Graphene as High-Performance Anode for Li-Ion Batteries. ACS Appl. Mater. Interfaces.

[34] Zhang H., van Pelt T., Mehta A.N., Bender H., Radu I., Caymax M., Vandervorst W., Delabie A. (2018). Nucleation and Growth Mechanism of 2D SnS_2_ by Chemical Vapor Deposition: Initial 3D Growth Followed by 2D Lateral Growth. 2D Mater..

[35] Kamkui H.M., Laminsi S., Njopwouo D., Djowe A.T. (2014). Deep Insight in Thermal Synthesis of Tin Disulphide (SnS_2_) Microplates, Starting from Tin Sulphate and Sulfur: Growth Mechanism based on LUX FLOOD’s Theory of Acid and Base. Chalcogenide Lett..

[36] Fu W., Wang J., Zhou S., Li R., Peng T. (2018). Controllable Fabrication of Regular Hexagon-Shaped SnS_2_ Nanoplates and Their Enhanced Visible-Light-Driven H_2_ Production Activity. ACS Appl Nano Mater..

[37] Zhang Y., Yuan X., Wang Y., Chen Y. (2012). One-Pot Photochemical Synthesis of Graphene Composites Uniformly Deposited with Silver Nanoparticles and their High Catalytic Activity towards the Reduction of 2-Nitroaniline. J. Mater. Chem..

[38] Zhang Z., Zhao H., Fang J., Chang X., Li Z., Zhao L. (2018). Tin Disulfide Nanosheets with Active-Site-Enriched Surface Interfacially Bonded on Reduced Graphene Oxide Sheets as Ultra-Robust Anode for Lithium and Sodium Storage. ACS Appl. Mater. Interfaces.

[39] Zhang Y.C., Du Z.N., Li S.Y., Zhang M. (2010). Novel Synthesis and High Visible Light Photocatalytic Activity of SnS_2_ Nanoflakes from SnCl_2_· 2H_2_O and S Powders. Appl. Catal. B..

[40] Kim K.M., Kwak B.S., Kang S., Kang M. (2014). Synthesis of Submicron Hexagonal Plate-Type SnS_2_ and band gap-tuned Sn_1−_xTixS_2_ Materials and Their Hydrogen Production Abilities on Methanol/Water Photosplitting. Int. J. Photoenergy.

[41] Zhou T., Pang W.K., Zhang C., Yang J., Chen Z., Liu H.K., Guo Z. (2014). Enhanced Sodium-Ion Battery Performance by Structural Phase Transition from Two-Dimensional Hexagonal-SnS_2_ to Orthorhombic-SnS. ACS Nano..

[42] Kim T.J., Kim C., Son D., Choi M., Park B. (2007). Novel SnS_2_-Nanosheet Anodes for Lithium-Ion Batteries. J. Power Sources.

[43] Lin Y.T., Shi J.B., Chen Y.C., Chen C.J., Wu P.F. (2009). Synthesis and Characterization of Tin Disulfide (SnS_2_) Nanowires. Nanoscale Res. Lett..

[44] Gajendiran J., Rajendran V. (2011). Synthesis of SnS_2_ Nanoparticles by a Surfactant-Mediated Hydrothermal Method and Their Characterization. Adv. Nat. Sci: Nanosci. Nanotechnol..

[45] Seo J.W., Jang J.T., Park S.W., Kim C., Park B., Cheon J. (2008). Two-Dimensional SnS_2_ Nanoplates with Extraordinary High Discharge Capacity for Lithium Ion Batteries. Adv. Mater..

[46] Degrauw A., Armstrong R., Rahman A.A., Ogle J., Whittaker-Brooks L. (2017). Catalytic Growth of Vertically Aligned SnS/SnS_2_ p–n Heterojunctions. Mater. Res. Express..

[47] Cheng L., Li D., Dong X., Ma Q., Yu W., Wang X., Yu H., Wang J., Liu G. (2017). Synthesis, Characterization and Photocatalytic Performance of SnS Nanofibers and SnSe Nanofibers Derived from the Electrospinning-made SnO_2_ Nanofibers. Mater. Res. (AHEAD).

[48] Karuppasamy P., Sivasubramani V., Pandian M.S., Ramasamy P. (2016). Growth and Characterization of Semi-Organic Third Order Nonlinear Optical (NLO) Potassium 3, 5-dinitrobenzoate (KDNB) Single Crystals. RSC Adv..

[49] Mondal C., Ganguly M., Pal J., Roy A., Jana J., Pal T. (2014). Morphology Controlled Synthesis of SnS_2_ Nanomaterial for Promoting Photocatalytic Reduction of Aqueous Cr (VI) under Visible Light. Langmuir.

[50] Shiga Y., Umezawa N., Srinivasan N., Koyasu S., Sakai E., Miyauchi M. (2016). A Metal Sulfide Photocatalyst Composed of Ubiquitous Elements for Solar Hydrogen Production. Chem. Commun..

[51] Lin C., Zhu M., Zhang T., Liu Y., Lv Y., Li X., Liu M. (2017). Cellulose/SnS_2_ Composite with Enhanced Visible-Light Photocatalytic Activity Prepared by Microwave-Assisted Ionic Liquid Method. RSC Adv..

[52] Li M., Liu E., Hu H., Ouyang S., Xu H., Wang D. (2014). Surfactant-Free Synthesis of Single Crystalline SnS_2_ and Effect of Surface Atomic Structure on the Photocatalytic Property. Int. J. Photoenergy.

[53] Liu H., Liu Y., Wang Z., He P. (2010). Facile Synthesis of Monodisperse, Size-Tunable SnS Nanoparticles Potentially for Solar Cell Energy Conversion. Nanotechnology.

[54] Liu G., Li Z., Hasan T., Chen X., Zheng W., Feng W., Jia D., Zhou Y., Hu P.A. (2017). Vertically Aligned Ultrathin SnS_2_ Nanosheets with Strong Photon Capturing Capability for Efficient Photoelectrochemical Water Splitting. J. Mater. Chem. A.

[55] Seminovski Y., Palacios P., Wahnón P. (2013). Effect of Van Der Waals Interaction on the Properties of SnS_2_ Layered Semiconductor. Thin Solid Films.

[56] Shown I., Samireddi S., Chang Y.C., Putikam R., Chang P.H., Sabbah A., Fu F.Y., Chen W.F., Wu C.I., Yu T.Y., Chung P.W. (2018). Carbon-Doped SnS_2_ Nanostructure as a High-Efficiency Solar Fuel Catalyst Under Visible Light. Nat. Commun..

[57] Pałosz B., Pałosz W., Gierlotka S. (1985). Polytypism of Crystals of Tin Disulphide; Structures of 21 Polytypes of SnS_2_. Acta Crystallogr. C.

[58] Ren W., Zhang H., Guan C., Cheng C. (2018). SnS_2_ Nanosheets Arrays Sandwiched by N-Doped Carbon and TiO_2_ for High-Performance Na-Ion Storage. Green Energy Environ..

[59] Huang P.C., Shen Y.M., Brahma S., Shaikh M.O., Huang J.L., Wang S.C. (2017). SnSx (x = 1, 2) Nanocrystals as Effective Catalysts for Photoelectrochemical Water Splitting. Catalysts.

[60] Soltani T., Entezari M.H. (2013). Photolysis and Photocatalysis of Methylene Blue by Ferrite Bismuth Nanoparticles Under Sunlight Irradiation. J. Mol. Catal. A Chem..

[61] Bhati A., Singh A., Tripathi K.M., Sonkar S.K. (2016). Sunlight-Induced Photochemical Degradation of Methylene Blue by Water-Soluble Carbon Nanorods. Int. J. Photoenergy.

[62] Wu Z., Wang F., Zuo S., Li S., Geng B., Zhuo R., Yan P. (2015). 3D Flower-Like Hierarchitectures Constructed by SnS/SnS_2_ Heterostructure Nanosheets for High-Performance Anode Material in Lithium-Ion Batteries. J. Nanomater..

[63] Li B., Xing T., Zhong M., Huang L., Lei N., Zhang J., Li J., Wei Z. (2017). A Two-Dimensional Fe-Doped SnS_2_ Magnetic Semiconductor. Nat. Commun..

[64] Tiwari J.N., Tiwari R.N., Kim K.S. (2012). Zero-Dimensional, One-Dimensional, Two-Dimensional and Three-Dimensional Nanostructured Materials for Advanced Electrochemical Energy Devices. Prog. Mater. Sci..

[65] Gong Y., Yuan H., Wu C.L., Tang P., Yang S.Z., Yang A., Li G., Liu B., van de Groep J., Brongersma M.L., Chisholm M.F. (2018). Spatially Controlled Doping of Two-Dimensional SnS_2_ through Intercalation for Electronics. Nat. Nanotechnol..

