# Supplementary material for: Photochemical Synthesis of Nanosheet Tin Di/Sulfide with Sunlight Response on Water Pollutant Degradation
Source: Nanomaterials (Basel). 2019 Feb 14;9(2):264. doi: 10.3390/nano9020264 (PMC6410158; doi:10.3390/nano9020264)
Supplement: Supplementary file 1 [file nanomaterials-09-00264-s001.docx]

Supplementary materials

Photochemical Synthesis of Nanosheets Tin Disulfide with Sunlight-Response on Water Pollutant Degradation

Juan Matmin ^1,^*, Mohamad Azani Jalani^2^, Hazwanee Osman ^1^, Qistina Omar ^1^, NorulNazilah Ab’lah^1^, Kelimah Elong^3^, and Firdaus Kasim^3^

| 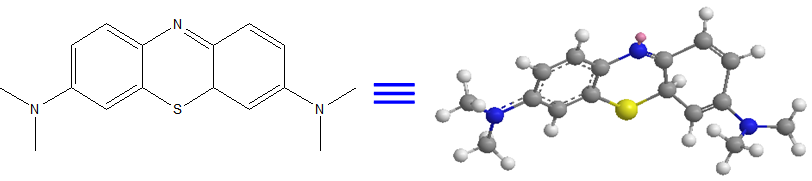 |  |
| --- | --- |

**Figure S1.** Methylene blue (MB) structures with chemical formula of C_16_H_18_N_3_S without the presence of Cl^-^ to simplify the structures.


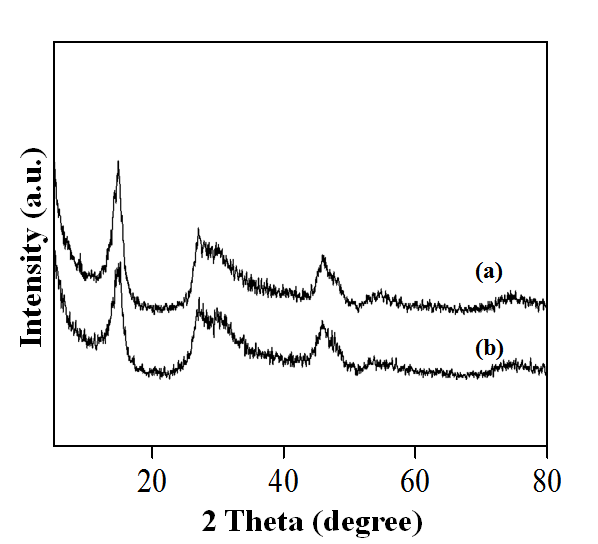


**Figure S2.** XRD patterns for tin di/sulfides (SnS_2-x_) prepared on different irradiation at **(a)** 30 W and **(b)** 60 W.


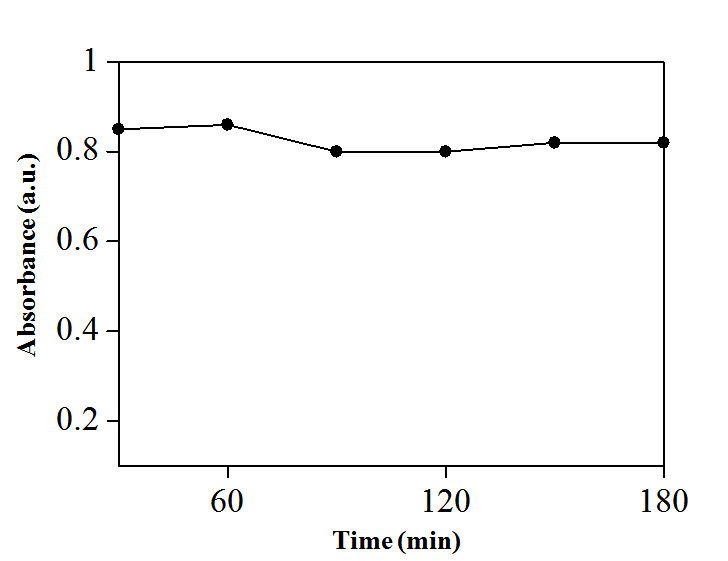


**Figure S3.** Adsorption-desorption equilibrium in dark condition of PS-SnS_2-x_ on MB for 180 min.

**
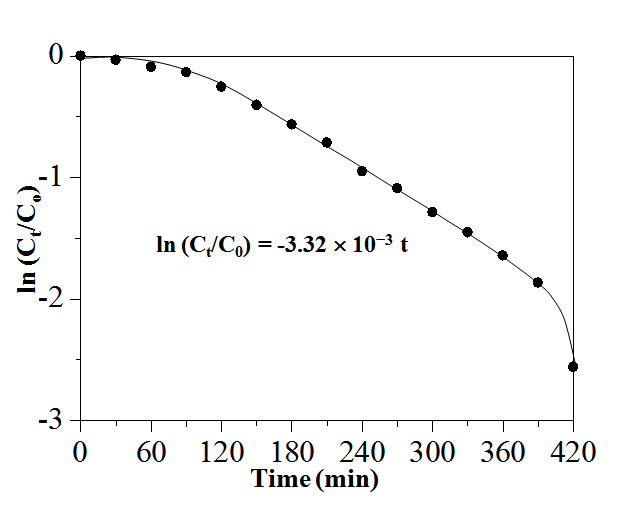
**

**Figure S4.** Plot on concentration of MB as a function of irradiation time for 180 min
